# WHEN AND WHERE: LESSONS LEARNED ABOUT TECHNOLOGY FROM AN INTERVENTION FOR CAREGIVERS OF PERSONS WITH HEART FAILURE

**DOI:** 10.1093/geroni/igac059.828

**Published:** 2022-12-20

**Authors:** Martha Abshire Saylor, Catherine Clair, Noelle Pavlovic, Katie Nelson, Lyndsay DeGroot, Janiece Taylor, Sarah Szanton

**Affiliations:** Johns Hopkins University School of Nursing, Baltimore, Maryland, United States; Johns Hopkins Bloomberg School of Public Health, Baltimore, Maryland, United States; Johns Hopkins University School of Nursing, Baltimore, Maryland, United States; Johns Hopkins University, Baltimore, Maryland, United States; Johns Hopkins University School of Nursing, Baltimore, Maryland, United States; Johns Hopkins University, Baltimore, Maryland, United States; Johns Hopkins University School of Nursing, Baltimore, Maryland, United States

## Abstract

Use of technology in older adult populations is growing, therefore it is important to understand opportunities for healthcare initiatives that support older adults using technology. The aim of the pilot study was to test Caregiver Support, a self-care and social support intervention, for caregivers of persons living with heart failure (N=24). Originally, the protocol was designed with in-person visits. We expected this option to reduce participant burden: the caregiver would not have to travel, and the interventionist would gain more insight about the home context to aid with intervention delivery. However, due to the COVID-19 pandemic, it became necessary to conduct the visits virtually. All participants completed the 5-component intervention via virtual meeting and there were no dropouts related to technology use. When asked about the virtual modality, participants emphasized the flexibility of virtual meetings. In summary, the intervention visits conducted virtually were perceived as a caregiver-centered approach.

